# Magnetoresistance manipulation and sign reversal in Mn-doped ZnO nanowires

**DOI:** 10.1038/srep35036

**Published:** 2016-10-14

**Authors:** Keshab R. Sapkota, Weimin Chen, F. Scott Maloney, Uma Poudyal, Wenyong Wang

**Affiliations:** 1 Department of Physics and Astronomy, University of Wyoming, Laramie WY, USA

## Abstract

We report magnetoresistance (MR) manipulation and sign reversal induced by carrier concentration modulation in Mn-doped ZnO nanowires. At low temperatures positive magnetoresistance was initially observed. When the carrier concentration was increased through the application of a gate voltage, the magnetoresistance also increased and reached a maximum value. However, further increasing the carrier concentration caused the MR to decrease, and eventually an MR sign reversal from positive to negative was observed. An MR change from a maximum positive value of 25% to a minimum negative value of 7% was observed at 5 K and 50 KOe. The observed MR behavior was modeled by considering combined effects of quantum correction to carrier conductivity and bound magnetic polarons. This work could provide important insights into the mechanisms that govern magnetotransport in dilute magnetic oxides, and it also demonstrated an effective approach to manipulating magnetoresistance in these materials that have important spintronic applications.

Dilute magnetic semiconductors (DMS) such as transition metal-doped metal oxides possess both ferromagnetic and semiconductor properties and are promising materials for the fabrication of spin field effect transistors (spin-FETs) in which charge and spin signals can be manipulated by external electric field^1^,^2^. This inclusion of the spin degree of freedom and its manipulation in FETs could create a wide range of potential applications in the next generation device technologies^3^. Several approaches have been investigated in order to achieve an efficient control over magnetotransport in DMS structures, such as altering the magnetic anisotropy energy or modulating the Rashba-Dresselhaus spin-orbit coupling; however, successful realization of spin signal manipulation still remains as a central problem in the field^4^,^5^. In this work we studied Mn-doped ZnO (Mn-ZnO) nanowires, an important one-dimensional DMS material, and explored an approach of utilizing gate-induced carrier concentration modulation to realizing magnetotransport manipulation in the device structures.

Research efforts have been devoted to the studies of metal oxide-based DMS materials to gain a better understanding of their ferromagnetic and magnetotransport behaviors^6^,^7^,^8^,^9^. In particular, magnetotransport in undoped and transition metal-doped ZnO has been studied in the forms of bulk, thin films, and nanowires, and these studies reported either positive or negative magnetoresistance (MR) depending on the type of transition metal doping, material structure, measurement temperature, and even the method used to prepare the samples^9^,^10^,^11^,^12^,^13^,^14^,^15^. For example, a recent study reported a gate-tuning effect on spin exchange interactions and magnetoresistance in undoped, ferromagnetic ZnO nanowires^16^. For transition metal-doped ZnO materials, a study of Co-doped ZnO films reported MR variation with increasing temperature, which was associated with the s-d splitting effect at low temperatures and a field-suppressed weak localization effect at high temperatures^17^. The magnetoresistance of Mn-doped ZnO films has also been inspected and large positive MR was reported^11^,^13^. However, magnetoresistance measurements of Mn-doped ZnO nanowires thus far only reported positive MR at low temperatures^9^. In this study we characterized Mn-doped ZnO nanowires and observed manipulation of magnetoresistance in both positive and negative regimes when the carrier concentration in the nanowires was modulated through the application of a back gate bias. More importantly, an MR sign reversal from positive to negative was observed when the carrier concentration was increased to a certain value. The observed MR behavior was further modeled by considering the combined effects of the quantum correction to carrier conduction due to s-d splitting in the applied magnetic field and the contribution from bound magnetic polarons (BMPs).

## Results

### Characterization of Mn-ZnO nanowires

**Figure 1:**
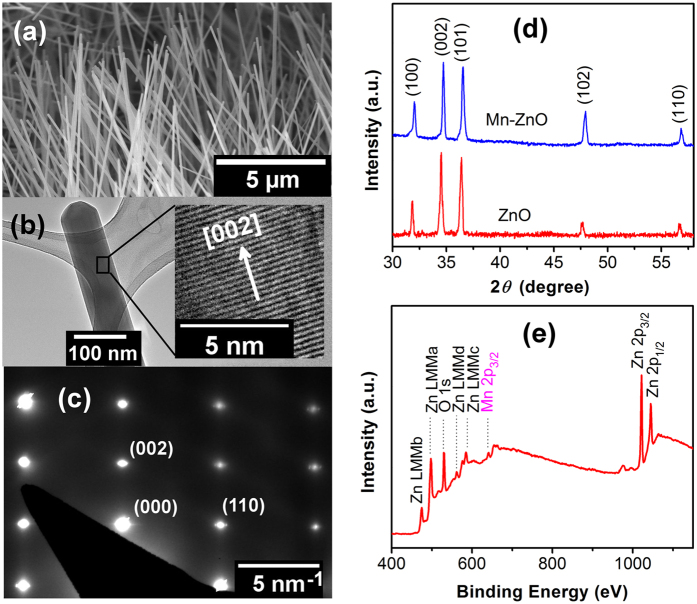
Mn-doped ZnO nanowires. (**a**) SEM image of as-grown Mn-ZnO nanowires. (**b**) Low magnification TEM image of a Mn-ZnO nanowire. The inset is a high-resolution TEM image showing the preferred [002] growth direction. (**c)** Selected area electron diffraction pattern of the Mn-ZnO nanowire. (**d**) XRD patterns of Mn-ZnO and ZnO nanowires. (**e**) A survey XPS spectrum of Mn-ZnO nanowires.

Figure 1(a) shows the Scanning Electron Micrograph (SEM) image of as-grown Mn-ZnO nanowires. The average nanowire diameter was ~100 nm and the average length was ~10 microns. These nanowires have a [002] preferred growth direction as shown in the Transmission Electron Microscopy (TEM) image in Fig. 1(b)^18^. Figure 1(c) shows the selected area electron diffraction pattern of the Mn-ZnO nanowire, revealing the single crystal structure of the nanowire. Figure 1(d) shows the X-ray diffraction (XRD) patterns of Mn-ZnO (upper) and ZnO (lower) nanowire films. The obtained XRD spectrum of Mn-ZnO nanowire film exhibited the wurtzite crystal structure, and no diffraction peaks corresponding to Mn precipitates or Mn-related secondary phases were detected within the XRD resolution limit, suggesting that Mn occupied the Zn sites in the doped nanowires^19^. X-ray photoelectron spectroscopy (XPS) measurement was also carried out to inspect the presence of Mn in the doped nanowires, and Fig. 1(e) shows the obtained XPS spectrum in which the peaks of Zn, O, and Mn were clearly identified. The peak at 640.7 eV was attributed to Mn 2p^3/2^, confirming that the Mn valence state in the doped nanowires was Mn^2+^ 9. From the XPS characterization the Mn doping concentration was estimated to be ~2 at. %, which was also confirmed by TEM Energy Dispersive X-ray Spectroscopy (EDX) measurement results (Supplementary Fig. S2).

**Figure 2:**
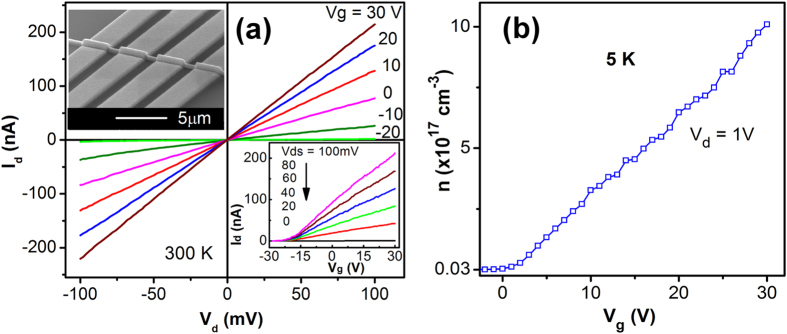
Electrical characterization of Mn-ZnO NWFETs. (**a**) *I*_*d*_*-V*_*d*_ characteristics at selected gate voltages at 300 K. Upper inset is the SEM image of a single-nanowire FET device. Lower inset shows the *I*_*d*_*-V*_*g*_ characteristics. (**b**) Carrier concentrations at different gate voltages in the Mn-ZnO NWFET at 5 K.

The upper inset in Fig. 2(a) shows the SEM image of a typical single Mn-ZnO nanowire field effect transistor (NWFET) with a channel length of ~1.5 μm and a nanowire diameter of ~100 nm. The NWFETs were characterized using the four-probe method to avoid contributions from the contact resistances. Figure 2(a) shows the room temperature *I*_*d*_*-V*_*d*_ measurement results of a NWFET at different gate voltages, which exhibited typical n-type FET characteristics. This n-type transport behavior of Mn-ZnO nanowires could be related to the presence of natural defects such as oxygen vacancies and Zn interstitials^20^,^21^. From the *I*_*d*_*-V*_*d*_ data the resistivity of the Mn-ZnO nanowire at *V*_*g*_ = 0 V was estimated to be *ρ* = 0.7 Ω∙cm. The electron mobility and concentration in the Mn-ZnO nanowire could be calculated from the *I*_*d*_*-V*_*g*_ characteristics that are presented in the lower inset in Fig. 2(a). The room temperature electron mobility was estimated to be 7.4 cm^2^/V∙s, while the electron concentration at *V*_*g*_ = 0 V was calculated to be 1.2 × 10^18^ cm^−3^ (details of the calculation is given in the Supplementary Information). Figure 2(b) shows the electron concentration in the Mn-ZnO NWFET at different gate voltages and *T* = 5 K, which were obtained from similar transport characterizations. It can be seen that at 5 K the electron concentration could be increased from 3.2 × 10^15^ to 1.0 × 10^18^ cm^−3^ when the gate voltage was increased from 0 to 30 V. The electron mobility at 5 K was calculated to be 12.5 cm^2^/V∙s from the transport data. The temperature-dependent resistance of the NWFET was characterized from 300 to 5 K (Supplementary Fig. S3), and when the temperature was decreased the nanowire resistance increased, exhibiting semiconducting (*dR/dT* < *0*) behavior as expected for Mn-ZnO nanowires.

### Magnetoresistance manipulation and sign reversal

**Figure 3:**
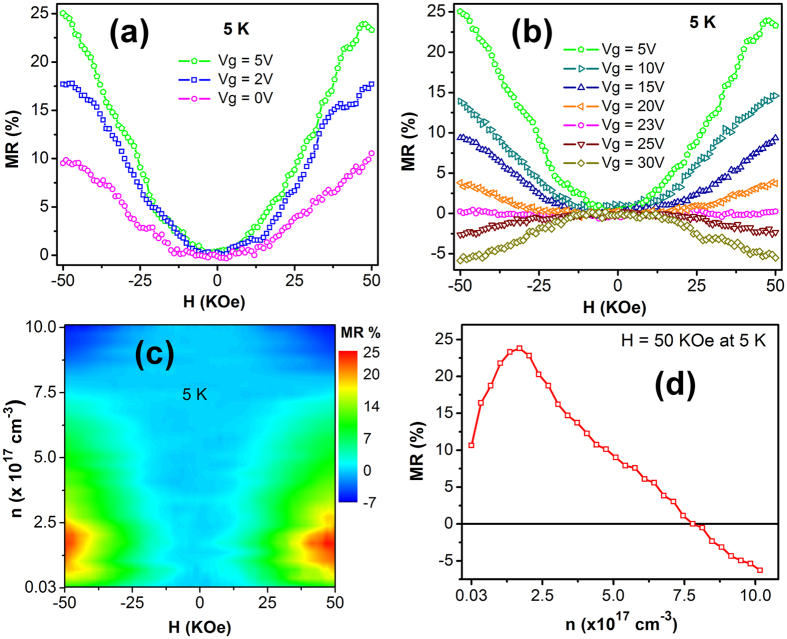
Magnetoresistance measurement results of the Mn-ZnO NWFET at different gate voltages and 5 K. *H* is perpendicular to the nanowire length axis. (**a**) Positive MR increased when *V*_*g*_ was increased from 0 to 5 V. (**b**) Positive MR decreased and exhibited a sign reversal when *V*_*g*_ was further increased. (**c**) Color plot of the MR as a function of carrier concentration and magnetic field. (**d**) MR as a function of carrier concentration at *H* = 50 KOe.

**Figure 4:**
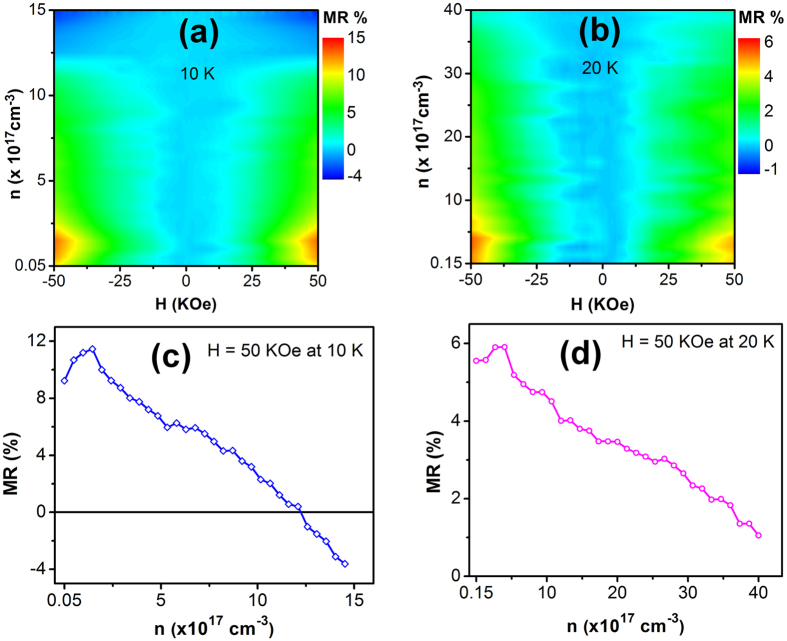
MR measurement results at 10 and 20 K. (**a**,**b**) Color plots of MR at 10 and 20 K, respectively. (**c**,**d**) MR as a function of carrier concentration at 50 KOe at 10 and 20 K, respectively.

Magnetoresistance of the Mn-ZnO NWFET was characterized at *T* = 5, 10, and 20 K. At 5 K, the NWFET threshold voltage was estimated to be ~1.3 V from the linear portion of the *I*_*d*_*-V*_*g*_ characteristics (Supplementary Fig. S4), thus the MR measurements were only carried out with positive gate voltages since a negative bias would deplete the conduction electrons and cause the current signal level to be lower than the equipment detection limit. Figure 3 shows the MR measurement results at different gate voltages and *T* = 5 K, where the magnetic field (*H*) was applied perpendicular to the nanowire length axis. It can be seen that the gate voltage had a strong effect on the magnetoresistance of the Mn-ZnO nanowire. As shown in Fig. 3(a), the nanowire initially exhibited positive MR at zero gate bias, and the MR percentage increased when *V*_*g*_ was increased from 0 to 5 V. When *V*_*g*_ became larger than 5 V, MR started to decrease but remained positive until the applied gate voltage reached 23 V as shown in Fig. 3(b). When *V*_*g*_ was further increased, the MR exhibited a sign reversal and became negative. The observed maximum positive MR was as high as 25% at *V*_*g*_ = 5 V and the minimum negative MR was 7% at *V*_*g*_ = 30 V. Since the applied gate voltage mainly modulated the carrier concentration in the Mn-ZnO NWFET, Fig. 3(c) shows the nanowire MR dependence on carrier concentration and applied magnetic field, while Fig. 3(d) shows the MR curve as a function of carrier concentration at *H* = 50 KOe. It can be seen from Fig. 3(d) that at 50 KOe the nanowire MR reached the maximum positive value at *n* = 1.7 × 10^17^ cm^−3^, while the transition from positive to negative MR occurred at *n* = 7.7 × 10^17^ cm^−3^. The MR measurement results at 10 and 20 K are presented in Fig. 4(a,b), respectively. At *H* = 50 KOe, clear positive MR maximum and MR sign reversal were still observed at *T* = 10 K [Fig. 4(c)]; however, at *T* = 20 K the positive MR maximum became less pronounced and the MR sign reversal was not detected in the measurement range [Fig. 4(d)]. In addition, when the temperature was increased from 5 to 20 K, the MR value was reduced while the occurrence of the positive MR maximum slightly shifted to a higher carrier concentration.

## Discussion

Several mechanisms have been proposed to explain the positive or negative magnetoresistance observed in dilute magnetic semiconductors, and depending on the transport regime different mechanisms could play different roles. One approach that is generally adopted to determine the transport regime is to calculate *k*_*F*_*λ*, where *k*_*F*_ is the Fermi wave vector, *λ* is the electron mean free path, and using the Drude model, *k*_*F*_*λ* = *ħ*(3*π*^2^)^2/3^/*ρe*^2^*n*^1/3^. When *k*_*F*_*λ* is less than 1 the carrier transport is believed to be in the localized regime^17^. Using our obtained carrier concentration and nanowire resistivity data, *k*_*F*_*λ* was estimated to be 0.054 at 300 K and *V*_*g*_ = 0 V, 0.002 at 5 K and *V*_*g*_ = 0 V, and 0.077 at 5 K and *V*_*g*_ = 30 V. Therefore, the carrier transport in the Mn-ZnO NWFET was expected to be in the localized regime in our measurement range. In this regime, one important factor that influences the magnetic field-dependent transport behavior is the spin-splitting of the conduction band induced by the s-d exchange interaction, which can cause a quantum correction to carrier conductivity and produce positive magnetoresistance^11^,^17^,^22^,^23^. On the other hand, bound magnetic polarons also play a role in magnetotransport in this localized regime. In Mn-doped ZnO, oxygen vacancies and Zn interstitials can act as shallow donors and provide electrons^24^,^25^,^26^. These hydrogenic electrons can become localized around the donor centers and interact with the 3d spins of the Mn^2+^ ions and form bound magnetic polarons^24^,^25^,^26^. The formation of BMPs in Mn-ZnO can affect the magnetotransport and produce negative magnetoresistance^13^,^27^,^28^. Therefore, the MR behavior of the Mn-ZnO NWFET observed in this study could be expected as a result of the competitions between these two factors. Specifically, at low carrier concentrations when the BMP population was not high, the contribution from the quantum correction dominated, which produced net positive magnetoresistance. However, when the BMP population became high enough at high carrier concentrations, its effect became more significant and could finally produce net negative magnetoresistance. There are also other mechanisms that could be involved in magnetoresistance changes in different systems, such as magnetic field-induced wave function shrinkage effect and spin scattering in a system of localized moments. However, the wave function shrinkage effect generally produces an exponential dependence of positive MR on low magnetic field, which was not observed in our measurements^29^. The spin scattering mechanism is only applicable to degenerate systems and can cause both positive and negative MR, but the detected MR typically saturates at moderate field (below 30 KOe as reported), which is different from what we observed in this study^16^,^30^. Other mechanisms such as weak localization and orbital quantum-interference effects can lead to negative MR. However, the weak localization mechanism is only applicable to systems with *k*_*F*_*λ* > 1, while the orbital quantum-interference effect is applicable only to weak field regime and usually produces MR saturation beyond 10 KOe^22^,^31^. Therefore, these mechanisms could not be used to model the MR behavior observed in this study, and instead we utilized the quantum correction and BMP models to fit our experimental data.

The magnetoresistance can be defined by *MR* = [*ρ*(*H*) − *ρ*(0)]/*ρ*(0). Considering the contributions from the quantum correction and BMPs, it can be further expressed as:


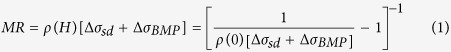


Δσ_*sd*_ in Equation (1) is the quantum correction to carrier conductivity in the presence of an external magnetic field due to s-d splitting of the disorder-modified electron-electron interaction, which is given by^17^,^22^:


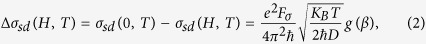



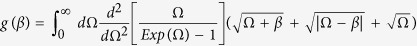


where *F*_*σ*_ is the Coulomb scattering screening parameter ranging from 0 to 1, *D* = *K*_*B*_*Tμ*/*e* is the diffusion coefficient with *μ* as the electron mobility, and 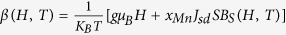 is the spin splitting term. Here 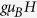 is from the Zeeman splitting and *x*_*Mn*_*J*_*sd*_*SB*_*S*_(*H*, *T*) describes the s-d splitting where *g* = 2, *x*_*Mn*_ = 0.02, *S* = 5/2, the s-d exchange energy *J*_*sd*_ = 0.2 eV, and *B*_*S*_(*H*, *T*) is the Brillouin function^32^. This quantum correction would produce positive MR and *F*_*σ*_ could be taken as a fitting parameter when this equation was used for MR fitting.

Δ*σ*_*BMP*_ in Equation (1) is associated with the contribution from BMPs. The BMPs can affect carrier transport because, due to magnetic coupling, they can increase the charge hopping barrier via lowering the system’s free energy by *Wp*(*H*, *T*), the polaron binding energy. When thermal fluctuation is not significant, the BMP hopping conductivity that is proportional to the BMP hopping rate between two sites is described by^24^,^33^


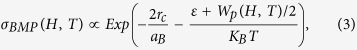


where *r*_*c*_ is the average distance between two hoping sites, *a*_*B*_ is the Bohr radius of hydrogenic electron and *ε* is the activation energy for the Millar-Abrahams hopping transport in the absence of BMPs. The polaron binding energy *W*_*p*_ (*H*, *T*) is given by^24^,^33^:


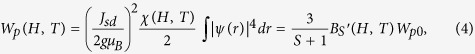


where *χ*(*H, T*) is the magnetic susceptibility and *W*_*p0*_ = *W*_*p*_ (*0*, *T*) is the zero field polaron binding energy. From Equation (4) it can be seen that an increase in the applied magnetic field would decrease the BMP binding energy via reducing the magnetic susceptibility, which could in turn increase σ_*BMP*_(*H*, *T*) and cause negative MR.

**Figure 5:**
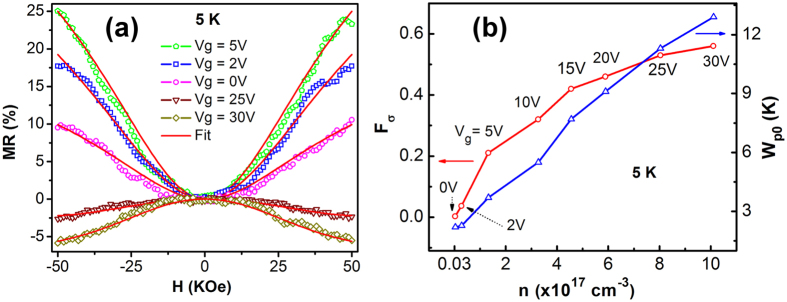
Fitting of the MR data at 5 K. (**a**) MR fitting at selected gate voltages. (**b**) Plots of the fitting parameters *F*_*σ*_ and *W*_*p0*_ at different carrier concentrations.

The above equations were used to fit the magnetoresistance data at 5 K with *F*_*σ*_ and *W*_*p0*_ as the fitting parameters. Figure 5(a) shows the fitting results at selected gate voltages, and Fig. 5(b) exhibits the obtained *F*_*σ*_ and *W*_*p0*_ values at different carrier concentrations. At zero gate bias the obtained values of *F*_*σ*_ and *W*_*p0*_ were 0.0028 and 2.2 K, respectively. After the application of the gate bias the carrier concentration in the Mn-ZnO NWFET was increased, so did *F*_*σ*_ and *W*_*p0*_. The increase in *F*_*σ*_ could be attributed to the enhanced Coulomb screening due to the fact that now there were more electrons interacting with the Coulomb force, while the increase in *W*_*p0*_ could be related to the increased *χ*(0, T) due to higher BMP population associated with increased carrier concentration^27^,^34^. As discussed previously, an increase in *F*_*σ*_ would cause the positive component of the magnetoresistance to increase, while an increase in *W*_*p0*_ would cause the negative component of the magnetoresistance to increase. When *V*_*g*_ was increased to 5 V, *F*_*σ*_ was increased by two orders of magnitude to 0.21 while *W*_*p0*_ was increased to 3.7 K. The effect of this initial rapid increase in *F*_*σ*_ suppressed that of the *W*_*p0*_ increase and produced a net positive increasing MR, which subsequently reached a maximum value. However, when the gate bias was further increased, the *W*_*p0*_ increase was almost linear while the *F*_*σ*_ increase became slower. From the equations above it can be seen that Δσ_*sd*_ depends on *F*_*σ*_ linearly while Δσ_*BMP*_ depends on *W*_*p0*_ exponentially, thus the combined effect caused the positive MR to decrease, and finally when the *W*_*p0*_ contribution to MR surpassed that from *F*_*σ*_ an MR sign reversal was observed. In addition, due to the small magnitude of *W*_*p0*_, the BMP contribution to MR was strongly affected by temperature [see Equation (3)]. This temperature effect on MR could be seen in Fig. 4, in which at 10 K the BMP contribution was still strong enough to cause an MR sign reversal while at 20 K the BMP contribution became significantly reduced and no MR sign reversal was detected in the carrier concentration range.

In summary, in this work we studied Mn-doped ZnO nanowires and explored magnetoresistance manipulation through the modulation of carrier concentration. At low temperatures positive MR was initially observed, and when the carrier concentration was increased the MR also increased and then reached a maximum value. However, further increasing the carrier concentration caused MR to decrease, and eventually an MR sign reversal from positive to negative was detected. The observed highest positive MR value was 25% and the lowest negative MR value was 7% at 5 K and 50 KOe. The MR behavior could be attributed to the combined effects of the quantum correction to carrier conductivity due to the s-d exchange interaction and the bound magnetic polarons. Specifically, at low carrier concentrations the magnetic field-dependent quantum correction to the diffusive transport was the dominant effect, which yielded positive MR. However, when the carrier concentration became higher, the increased BMP population started to play a dominant role, which caused decreased MR and eventually a sign reversal. This work could provide important insights into the investigation of mechanisms that govern magnetotransport in dilute magnetic oxides. It also demonstrated an effective approach to manipulating magnetotransport in DMS structures that could have important magnetic and spintronic applications.

## Methods

### Synthesis of Mn-ZnO nanowires

Mn-doped ZnO nanowires were synthesized via a chemical vapor deposition method using manganese chloride tetrahydrate (4H_2_O∙MnCl_2_) powder and zinc foil as a self-seeding layer. The source materials were placed on a silicon wafer and inserted into a horizontal 1″ quartz tube furnace, which was then evacuated to a base pressure of 1 mTorr. After several purges with argon gas, the pressure in the tube was throttled to 8 Torr under a constant flow of 20 sccm argon for processing. The temperature was then increased to 850 °C at a rate of 20 °C per minute. Upon reaching 420 °C, a constant flow of 50 sccm air was introduced into the tube. The pressure was adjusted accordingly to maintain 8 Torr during the reaction (Supplementary Fig. S1(a)).

### Device fabrication and measurement

Back-gated field effect transistors of single Mn-ZnO nanowires (Supplementary Fig. S1(b)) were fabricated by standard electron beam lithography and lift-off process. Specifically, Mn-ZnO nanowires were removed from the growth substrate using isopropyl alcohol and were dispersed on a clean SiO_2_/Si substrate. The substrate with Mn-ZnO nanowires was coated with polymethyl methacrylate (PMMA) and was baked at 180 °C for 2 minutes. E-beam lithography was then performed to define the electrode patterns. Before top metallization, the exposed portion of the nanowire was etched in diluted HCl for 5 seconds to clean the nanowire surface. Metallization was carried out in an ultra-high vacuum chamber, and Ti (5 nm) and Au (150 nm) were deposited to form the electrodes. To improve the nanowire/electrode contacts, the devices were annealed at 300 °C for 20 minutes. Transport properties of the single-nanowire FETs were characterized using Quantum Design Physical Properties Measurement System (PPMS) as well as Agilent Semiconductor Parametric Analyzer 4156.

## Additional Information

**How to cite this article**: Sapkota, K. R. *et al*. Magnetoresistance manipulation and sign reversal in Mn-doped ZnO nanowires. *Sci. Rep*. **6**, 35036; doi: 10.1038/srep35036 (2016).

## Supplementary Material

Supplementary Information
