# Supplementary material for: Magnetoresistance manipulation and sign reversal in Mn-doped ZnO nanowires
Source: Sci Rep. 2016 Oct 14;6:35036. doi: 10.1038/srep35036 (PMC5064367; doi:10.1038/srep35036)
Supplement: Supplementary Information [file srep35036-s1.pdf]

Supplementary Information *for*

Magnetoresistance manipulation and sign reversal in Mn-doped ZnO nanowires

Keshab R. Sapkota, Weimin Chen, F. Scott Maloney, Uma Poudyal, and Wenyong Wang

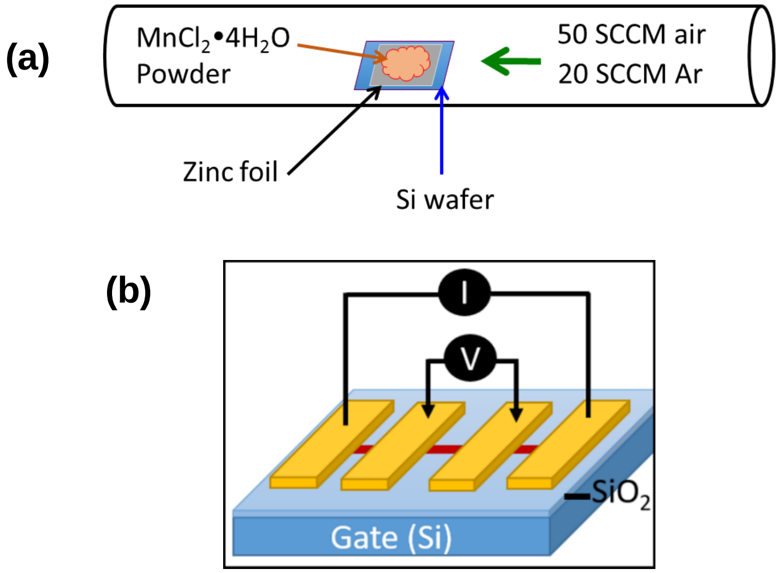

Figure S1: Mn-doped ZnO (Mn-ZnO) nanowires growth and device schematic. (a) Schematic of chemical vapor deposition (CVD) method to grow Mn-ZnO nanowires. (b) Four probe measurement scheme of the single nanowire field effect transistor (FET)

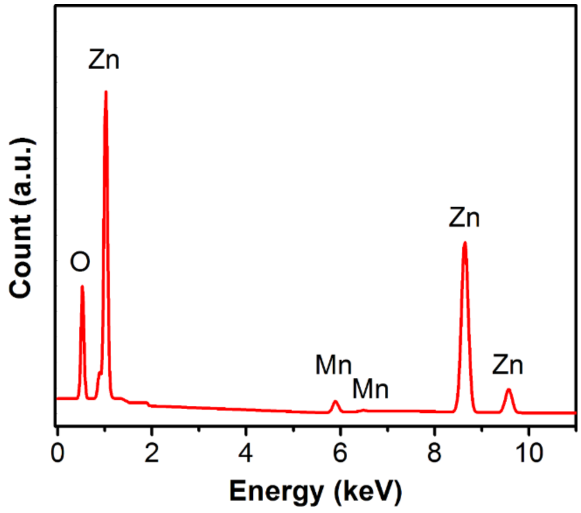

Figure S2: TEM-Energy-dispersive X-ray spectroscopy of single Mn-ZnO nanowire. The measurement exhibited the 2 at. % Mn on the nanowire

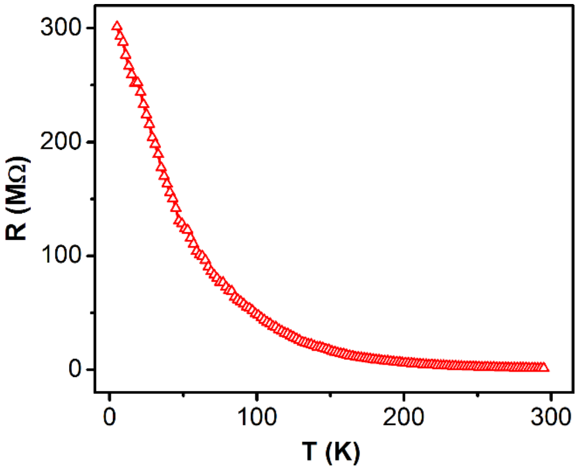

Figure S3. Temperature dependent resistance *(R-T)* measurement of the Mn-ZnO nanowire device at $V_g = 0$ V, which shows semiconductor behavior ($dR/dT < 0$) for entire measurement range of temperature.

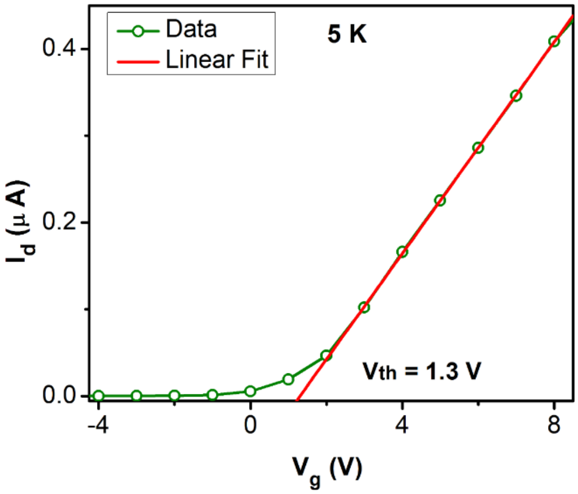

Figure S4. Determination of threshold gate voltage of nanowire FET at 5K. Fitting of linear portion of curve is done to estimate the threshold voltage V_{th}.

Calculation of mobility and carrier concentration of nanowire FET

The field effect electron mobility and carrier concentration of the Mn-ZnO nanowire has been extracted from the $I_d - V_g$ measurements as shown in Figure 2. The room temperature resistivity

of Mn-ZnO nanowire at V_g = 0V is estimated to be ρ = 0.7 Ω·cm. The mobility μ can be estimated from the equation:

$$\mu = \frac{g_m l^2}{V_d C_{ox}}; \;\; g_m = dI_d/dV_g$$

, where g_m is transconductance which is obtained from the slope of I_d –V_g curve above threshold voltage, l = 1.5 µm is nanowire channel length, V_d is drain to source voltage corresponding to g_m, and C_{ox} is the gate capacitance. For the cylindrical nanowire on the plain dielectric surface, C_{ox} can be obtained by the equation[1]:

$$C_{ox} = \frac{2\pi\epsilon_{ox}\epsilon_0 l}{Cosh^{-1}\left(1+\frac{2t_{ox}}{d}\right)}$$

, where $\epsilon_0 = 8.85 \times 10^{12}\, F/m$ is absolute permittivity, $\epsilon_{ox} = 3.9$ is the dielectric constant of the SiO_2, t_{ox} = 300 nm is the SiO_2 layer thickness and d = 110 nm is the diameter of Mn-ZnO nanowire. Room temperature transconductance g_m = 4.2 × 10^{-9} S is evaluated at V_d = 100 mV in the region of Vg = 0 V where I_d –V_d curve is linear and slope is maximum. The gate capacitance is calculated to be C_{ox} = 1.274 × 10^{-16} F. Using C_{ox} and g_m, room temperature field effect electron mobility is obtained as μ = 7.4 cm^2 .V^{-1}.s^{-1}. The room temperature electrons carrier concentration n of the Mn-ZnO nanowire at Vg = 0 is obtained as n = 1.2 × 10^{18} cm^{-3} by using the equation: $n = 1/\rho e\mu$ where e is electronic charge.

References

1. Wunnicke, O. Gate capacitance of back-gated nanowire field-effect transistors. *Appl. Phys. Lett.* **89,** 083102 (2006).
